# IntroSpect: Motif-Guided Immunopeptidome Database Building Tool to Improve the Sensitivity of HLA I Binding Peptide Identification by Mass Spectrometry

**DOI:** 10.3390/biom12040579

**Published:** 2022-04-14

**Authors:** Le Zhang, Geng Liu, Guixue Hou, Haitao Xiang, Xi Zhang, Ying Huang, Xiuqing Zhang, Bo Li, Leo J. Lee

**Affiliations:** 1 College of Life Sciences, University of Chinese Academy of Sciences, Beijing 100049, China; zhangle2@genomics.cn (L.Z.); xianghaitao@genomics.cn (H.X.); zhangxq@genomics.cn (X.Z.); 2 BGI-GenoImmune, BGI-Shenzhen, Wuhan 430074, China; liugeng@genomics.cn (G.L.); huangying3@genomics.cn (Y.H.); 3 BGI-Shenzhen, Shenzhen 518083, China; houguixue@bgi.com (G.H.); zhangxi1@cngb.org (X.Z.); 4 Department of Electrical and Computer Engineering, Donnelly Centre for Cellular and Biomolecular Research, University of Toronto, Toronto, ON M5S 3G4, Canada

**Keywords:** immunopeptidome, mass spectrometry, database search, motif, search space

## Abstract

Although database search tools originally developed for shotgun proteome have been widely used in immunopeptidomic mass spectrometry identifications, they have been reported to achieve undesirably low sensitivities or high false positive rates as a result of the hugely inflated search space caused by the lack of specific enzymic digestions in immunopeptidome. To overcome such a problem, we developed a motif-guided immunopeptidome database building tool named IntroSpect, which is designed to first learn the peptide motifs from high confidence hits in the initial search, and then build a targeted database for refined search. Evaluated on 18 representative HLA class I datasets, IntroSpect can improve the sensitivity by an average of 76%, compared to conventional searches with unspecific digestions, while maintaining a very high level of accuracy (~96%), as confirmed by synthetic validation experiments. A distinct advantage of IntroSpect is that it does not depend on any external HLA data, so that it performs equally well on both well-studied and poorly-studied HLA types, unlike the previously developed method SpectMHC. We have also designed IntroSpect to keep a global FDR that can be conveniently controlled, similar to a conventional database search. Finally, we demonstrate the practical value of IntroSpect by discovering neoepitopes from MS data directly, an important application in cancer immunotherapies. IntroSpect is freely available to download and use.

## 1. Introduction

The study of immunopeptidome, which is the collection of peptides presented on a cell surface by major histocompatibility complex (MHC) molecules, is invaluable to the development of next-generation vaccines and immunotherapies against autoimmunity, infectious diseases and cancer [1,2,3,4,5,6,7,8]. Usually, the identification of immunopeptidome by mass spectrometry (MS) is carried out with standard database search tools [9,10], such as MS-GF+ [11], Comet [12], X!Tandem [13] and MaxQuant [14]. These tools, however, originally tailored to shotgun proteome, may bring a risk of low sensitivity when used in immunopeptidome [15,16,17]. In shotgun proteome, the proteins are treated by digestive enzymes in the experiment, and the cleavages that occur only at specific sites can significantly reduce the space available for the database search. When it comes to immunopeptidome, the proteins are not digested in the experiment, but are digested by proteasome in the cells with non-specific cleavages, resulting in a huge search space [18,19,20,21,22,23]. Previous studies have confirmed that the overly inflated search space will reduce the statistical power and sensitivity in database search [24,25,26].

Conventional database search for immunopeptidome consists of the following steps: generating search space by unspecific digestion, assigning the spectra of MHC-bound peptides to their sequences and scoring and filtering assignments by a certain false discovery rate (FDR) [27,28]. In order to increase the sensitivity of immunopeptidome database search, two classes of computational methods have been developed: the first class, including MSrescue [21], DeepRescore [22] and MHCquant [29], aims to optimize the scoring and filtering of assignments, and will be referred to as post-processing tools in this manuscript; the second class, SpectMHC [18], aims to optimize the generation of search space, and will be referred to as database building tools. SpectMHC builds the targeted search space based on HLA-peptide binding predictions, which is trained from existing HLA-binding peptide databases. Its performance will be heavily influenced by the accuracy of the corresponding binding prediction, which may not work well for poorly-studied HLAs [21]. Furthermore, SpectMHC combines the iterative searches of unspecific digestion database and HLA-binding peptide database, making it infeasible to calculate a global FDR [30], which is important for controlling the overall error rate [31].

Here we developed a novel motif-guided immunopeptidome database building tool named IntroSpect to increase the sensitivity of immunopeptidome detection. IntroSpect trains data-efficient PSSM models based on the high scoring peptides identified by conventional database search and builds a targeted database to carry out refined search. In the remainder of this paper, we will detail the development of IntroSpect, demonstrate its superior performance over existing database building tools and show how it can be used to identify neoepitopes from MS data directly. We believe our freely available, open-source tool makes a valuable contribution to advance the field of immunopeptidomics.

## 2. Materials and Methods

### 2.1. Generation of Cell Lines

The K562 and HCT116 cell lines were obtained from ATCC (American Type Culture Collection, Manassas, VA, USA), and the K562 cell line was engineered to express a single HLA-allele as described previously [32]. In short, it was transduced using a highly efficient retroviral vector coding HLA-A*11:01. The vectors were transfected into a 293T packaging cell line, and replication-defective virus supernatants were harvested. After infection of K562 cells with the supernatant, antibody-directed flow cytometry sorting was done to obtain cells with high expressions of HLA-A*11:01. Cells were grown in T75 flasks to a density of 1 × 10^9^ cells before harvesting for experiments.

### 2.2. Purification of HLA-I Peptides

HLA-I peptides were obtained from K562 and HCT116 cells as described previously [33]. In brief, 1 × 10^9^ cells were dissociated using 40 mL of lysis buffer with 0.25% Sodium deoxycholate, 1% n-octyl glucoside, 100 mM PMSF and protease inhibitor cocktails in PBS at 4 °C for 60 min. Lysate were further cleared by 30 min centrifugation at 14,000× *g*. Cleared lysate were immunoaffinity purified with pan-HLA class I complexes antibody covalently bound to Protein-A Sepharose CL-4B beads. Beads were first washed with 10 column volumes of 150 mM NaCl, 20 mM Tris HCl (buffer A), then 10 column volumes of 400 mM NaCl, 20 mM Tris HCl, then 10 volumes of buffer A again, and finally with 10 column volumes of 20 mM Tris HCl, pH 8.0. The HLA-I molecules were eluted at room temperature using 0.1 N acetic acid. Eluate were then loaded on Sep-Pak tC18 cartridges (Waters, 50 mg) and washed with 0.1% TFA. The peptides were separated from HLA-I complexes on the C18 cartridges by eluting with 30% ACN in 0.1% TFA and concentrated to 20 µL using vacuum centrifugation. Finally, a 5 µL sample was used for MS analysis.

### 2.3. LC-MS/MS Analysis of HLA-I Peptides

HLA-I peptides of K562 and HCT116 cells were separated by HPLC (15 cm-long, 75 µm inner diameter columns with ReproSil-Pur C18-AQ 1.9 µm resin) and eluted into an Orbitrap Fusion Lumos mass spectrometer (Thermo Fisher Scientific, Waltham, MA USA). Peptides were separated with a gradient of 2–30% buffer (80% ACN and 0.5% acetic acid) at a flow rate of 250 nL/min over 65 min. MS was performed using data-dependent acquisition (DDA) mode. MS1 scans were conducted at a resolution of 120,000 over a scan range of 350–1500 *m*/*z* with a target value of 3 × 10^6^. Based on MS1 scans, MS2 scans were conducted at a resolution of 60,000 at 100 *m*/*z* with a target value of 1 × 10^5^. Fragment ion was produced by higher energy collisional dissociation (HCD) at 28% collision energy with a precursor isolation window of 2 *m*/*z*.

### 2.4. Sequencing and Analysis

For HCT116 cell line, DNA extractions, libraries construction and sequencing (pair-end 100 bp) were conducted according to protocols of MGISEQ-2000 platform (BGI-Shenzhen, China). RNA-Seq data of the HCT116 cell line were downloaded from the NCBI (SRR4228899). Low-quality reads were removed with SOAP nuke [34] v1.5.6. DNA-Seq data were processed by minimap2 [35] v2.11 for read alignment and GATK [36] v3.7.0 for variant analysis. RNA-Seq data were processed by HISAT [37] v2.1.0 for read alignment, GATK [36] v3.7.0 for variant analysis and RSEM [38] v1.3.0 for transcript quantification. Mutations were called with respect to the reference genome and those with more than 1% population frequency in dbSNP databases were removed, resulting in a total of 6220 SNVs (single nucleotide variants) and 1679 INDELs (insertions and deletions). A total of 480,905 potential neoepitopes (9–11 mers) were generated from the sequences with these SNVs and INDELs.

### 2.5. Mass Spectrometry Database Search

The remaining MS/MS datasets were downloaded from public databases (B721.221, MSV000080527 [39] in MassIVE; Train1~Train63, MSV000082648 [40] in MassIVE; Jurkat, PXD011723 [21] in PRIDE). The raw files of public and inhouse MS data were converted to mgf files using ProteoWizard msConvertGUI [41]. For the conventional search, the database contains 161,521 Uniprot [42] human protein entries (20 December 2017) and 245 frequently observed contaminants, such as human keratins, bovine serum proteins and proteases. Additionally, 480,905 potential neoepitopes mentioned earlier were added to the database when searching the HCT116 datasets. For IntroSpect search, the database contains the peptides that passed the filtering of PSSM models and the peptides identified by conventional search. For SpectMHC search, the database contains the peptides with BA rank score ≤2%, predicted by netMHCpan4.1. The MS-GF+ search tool (release 17 July 2018) was separately employed to search the above databases against the various MS datasets. Parameters of MS-GF+ are: variable modifications, N-terminal acetylation (42.010565 Da) and methionine oxidation (15.994915 Da); enzyme, unspecific cleavage (no cleavage for IntroSpect and SpectMHC search); precursor ion tolerance, 10 ppm; peptide length, 9–11; and charge, 2–5. The Percolator [43] (version 3.02.0) post-processing tool was applied for the estimation at the peptide level of <1% FDR after database search. From the pout.tab output file generated by Percolator, assignments to the contaminants were eliminated. The parameter settings of the MaxQuant and Comet search tools are consistent with those of MS-GF+ mentioned above.

### 2.6. Gibbs Clustering of HLA-I Peptides

The peptides identified by a conventional database search were clustered into various groups using GibbsCluster-2.0 Server [44], with the following parameters: number of clusters, 1–6; motif length, 9; max deletion length 2; max insertion length 0; number of seeds for initial conditions, 5; penalty factor for inter-cluster similarity, 0.8; weight on small clusters, 5; use trash cluster to remove outliers, enable; threshold for discarding to trash, 2; and number of iterations per sequence per temperature step, 10. The peptides in the clusters with the highest KLD were retained for further analysis.

### 2.7. PSSM Model Training and Filtering

Based on the clusters, we built PSSM models as described previously [45] to learn the corresponding sequence motifs for peptides in different groups. Briefly, each element *P_ai_* in the PSSM matrix is the likelihood of a specific amino acid *a* at a given position *i*. We calculated *P_ai_* as follows

$$P_{ai}=\log\frac{F_{ai}+\omega }{B_{a}},$$

where *F_ai_* denotes the frequency of a specific amino acid at the specific position in the peptides identified by conventional search; *B_a_* denotes the frequency of the specific amino acid from a background database (such as Uniprot human protein database); and *ω* is a value generated from a Dirichlet distribution [46] to avoid overfit, which is equivalent to adding a small number of ‘pseudo counts’ to the effective observations. To filter the whole proteome database to generate a targeted one, we define the motif score of a given peptide as the sum of the *P_ai_* at each site in the PSSM, and only kept those with a motif score greater than 0.3.

### 2.8. Synthetic Peptide Validation

A total of 118 randomly selected peptides from K562 dataset were synthesized and analyzed under the same MS conditions with K562 HLA I peptides. The mirror plots of spectra between synthetic peptides and eluted peptides were generated by PDV [47]. To validate a peptide which could be presented by MHC-I complex, the following criteria were considered: (i) the variation of retention time between precursor ions was less than 3 min; (ii) the pattern and retention time were matched between synthetic and target peptides with no less than 5 product ions.

### 2.9. Peptide Pearson Correlation Coefficient (PCC) Calculation

To quantify the similarity between two sets of peptides with the same length, we calculated the Pearson Correlation Coefficient (PCC) of the amino acid frequencies between them. For a given position *i*, we first calculated the empirical probability mass functions (pmfs) of the amino acid distributions in both the first (*x*) and second (*y*) sets. The PCC between these two random variables *X_i_* and *Y_i_*, *PCC_XiYi_*, is then computed as

$$PCC_{X_{i}Y_{i}}=\frac{cov\left(X_{i},Y_{i}\right)}{\sigma_{X_{i}}\sigma_{Y_{i}}},$$

where *cov* is the covariance and *σ*’s are the standard deviations.

### 2.10. Code Availability

We have made IntroSpect available on GitHub: https://github.com/BGI2016/IntroSpect (accessed on 9 April 2022). This is a command-line tool written in Perl, which requires GibbsCluster v2.0 preinstalled, in Darwin (Mac) or Linux platforms. The tool takes an input protein FASTA database and peptides identified by a conventional search and outputs targeted database which could be used for refined high-sensitivity identification.

## 3. Results

### 3.1. The Development of IntroSpect

In order to reduce the overly inflated search space caused by unspecific digestions, we adopted a strategy of motif-guided digestion in IntroSpect. The motif-guided digestion leads to a small and targeted database in which the peptides that are extremely unlikely to be present in a given sample will be filtered out. Peptides that do exist in the sample will obtain higher q values due to less competition, making it easier for real peptides to stay after FDR filtering. Therefore, IntroSpect can achieve higher statistical power and identify more peptides at the same FDR.

**Figure 1:**
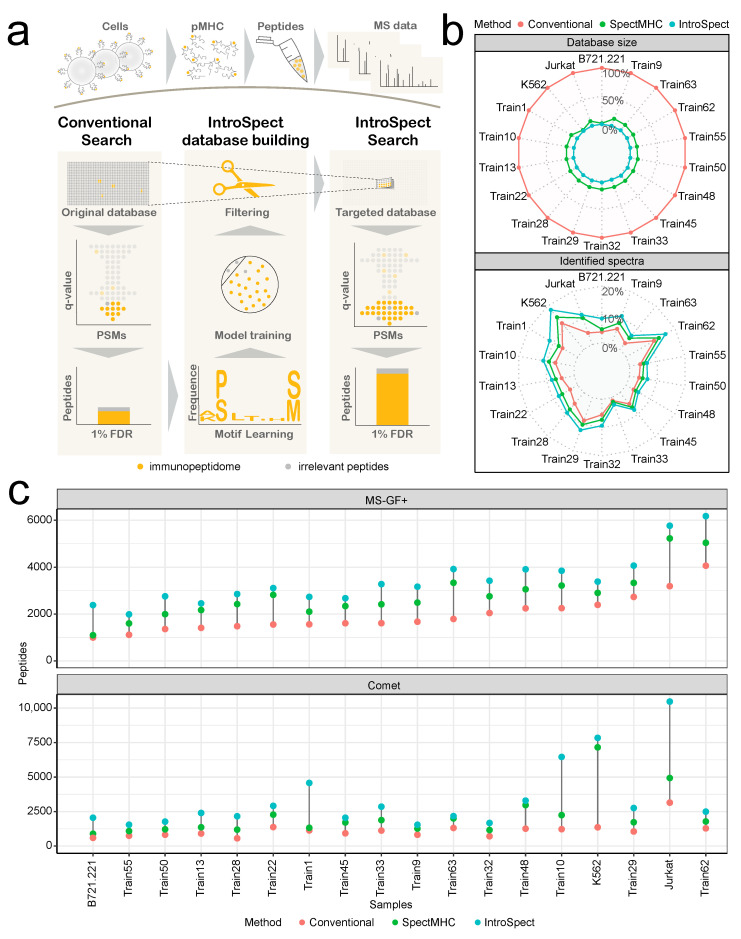
IntroSpect improves peptide identification sensitivity by reducing the search space. (**a**) The flowchart of the conventional database search and IntroSpect database search. (**b**) IntroSpect and SpectMHC decreased the database size and increased the proportion of identified MS/MS spectra with MS-GF+. The database size is calculated as the number of 9–11 mer peptides in the database. (**c**) IntroSpect and SpectMHC significantly increased the identified peptides with MS-GF+ and Comet search engines, while IntroSpect consistently outperformed SpectMHC in terms of sensitivity.

Searching with IntroSpect includes four steps (Figure 1a). Step 1 is to import the conventional protein database and MS raw data into the search engine and obtain peptides that pass 1% FDR filtering. These high-confidence peptides are then clustered into groups by GibbsCluster2.0 in step 2, and peptides in the same group are used to train a position-specific scoring matrix (PSSM) model to learn their motifs. In step 3, the PSSM model is used to score each peptide in the conventional database and peptides with PSSM score > 0.3 (the default threshold of IntroSpect), as well as those with FDR < 1% in the first round, are combined to become the new search space. Step 4 runs the second-round search against this new, targeted database to identify peptides that pass 1% FDR as the final output. Unlike previous multi-round search strategies [18,19,20] where different rounds of results are combined, we decide to add the first-round peptides directly into the targeted database for the second (and final) round search, so that a global FDR can be obtained. As we will show later, the vast majority of first round peptides will still appear in the final results. Steps 2 and 3 are written in Perl to form the IntroSpect package, while steps 1 and 4 are left to the users to decide how to build the conventional database and to run the search engine of their choice. Users can also adjust the threshold of PSSM Score or the range of peptide lengths to make IntroSpect suitable for different experiments.

### 3.2. IntroSpect Can Identify Substantially More Peptides

To evaluate IntroSpect, we tested its performance on 18 MHC class I immunopeptidome datasets (Table 1). In order to facilitate the comparison of different data sets, only 9–11 mer peptides were analyzed in the test [48,49]. We first used MS-GF+ as the search engine, in tandem with Percolator for 1% FDR filtering and ran IntroSpect, SpectMHC and conventional database search to identify peptides on these datasets. The databases generated by IntroSpect are much smaller than the conventional database, accounting for only 0.52 to 3.16% of the latter (Figure 1b), and are also considerably smaller than those generated by SpectMHC (2.22 to 14.75%). Moreover, the IntroSpect database search resulted in higher proportions of identified spectra (2.65% to 18.22%) than the conventional database search (1.11 to 12.07%) and SpectMHC database search (1.61% to 14.81%) under the same FDR (Figure 1b). The improvements on the number of identified peptides were even more significant: on average, IntroSpect identified 76.50% more peptides than conventional search (*p* = 1.1 × 10^−5^, the Wilcoxon test) and 23.17% more than SpectMHC (*p* = 0.04) (Figure 1c). Similar results were obtained when testing on Comet (Figure 1c and Figure S1), another popular search engine: IntroSpect identified 200.00% (*p* = 2.6 × 10^−6^) more peptides than those of conventional search and 87.61% more than SpectMHC (*p* = 0.0082) on average. We also tested IntroSpect on 3 of the 18 datasets with MaxQuant, and it can identify 98.01% more peptides than those of conventional database search and 87.16% more than SpectMHC (Figure S2). As expected, we observed that the identified peptides obtained higher MS/MS scores and lower q-values in general due to the reduction of irrelevant peptides (Figures S3 and S4). In order to focus on the differences between IntroSpect, SpectMHC and the conventional search, the results in the following sections were obtained by using only one search tool (MS-GF+).

### 3.3. IntroSpect Achieved a Similar Accuracy as Conventional Search

**Figure 2:**
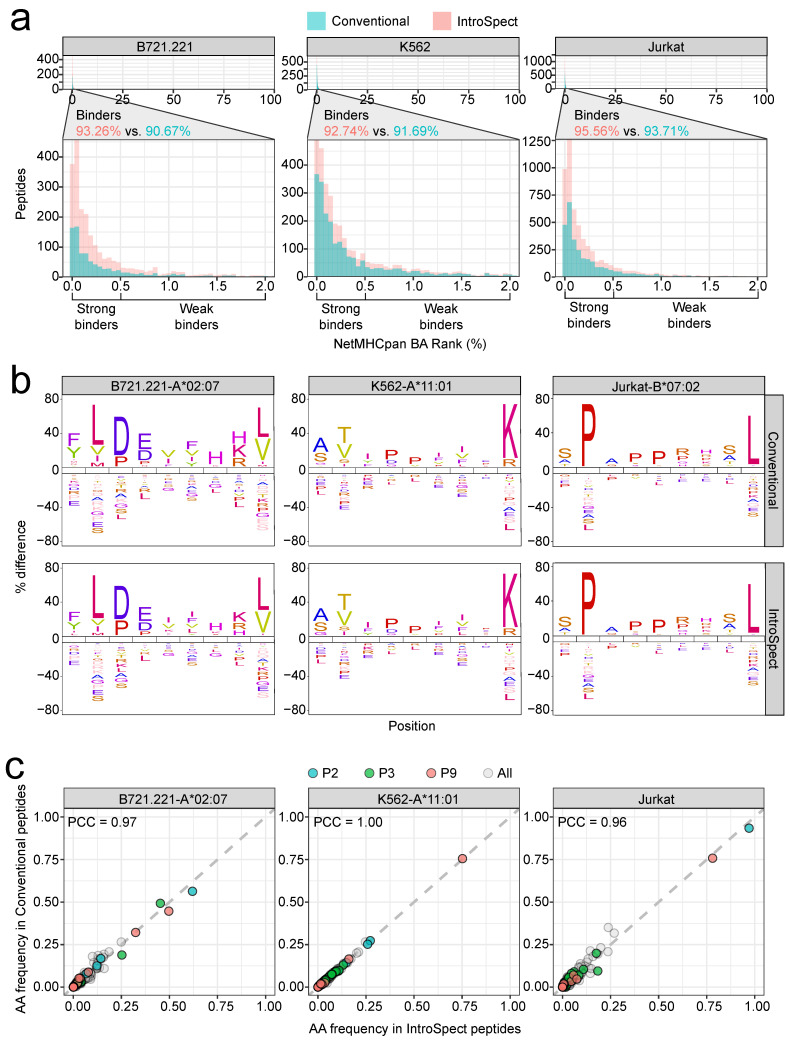
Immunopeptides from IntroSpect and conventional database search are very similar. (**a**) The histogram of predicted BA rank values of peptides identified by conventional and IntroSpect search: the peptides in separate panels are predicted to be strong or weak binders (BA rank < 2%), with their percentages marked on the panel. (**b**) The sequence logos of immunopeptides in three datasets (B721.221-A*02:07, K562-A*11:01 and Jurkat-B*07:02) identified by the conventional and IntroSpect search. (**c**) Amino acid frequencies at each position for peptides identified by the conventional and IntroSpect search.

Here we focused on results obtained from MS-GF+ on 3 of the 18 datasets, B721.221, K562 and Jurkat, to analyze the accuracy of peptides identified by IntroSpect, while results on more datasets and from other search engines are shown in the Supplementary Material (Figures S5–S8). We first compared the proportion of identified peptides predicted to be binders by both IntroSpect and conventional search, a strategy that has been previously applied to check for the quality of MS data [21,22,39,50]. We predicted the binding affinity (BA) rank of peptides using netMHCpan 4.0 [51], and drew the histogram of BA rank values for all identified peptides, with a zoomed-in panel for binders (BA rank < 2.0%, Figure 2a). Note that SpectMHC was not included in this analysis, since netMHCpan has already been used when building the targeted database. For all three datasets, most peptides identified by both IntroSpect and conventional search were predicted as binders, and the overall distribution is quite similar, with those identified by IntroSpect having slightly more binders (95.56 vs. 93.71% for Jurkat, 92.74 vs. 91.69% for K562 and 93.26 vs. 90.67% for B721.221).

HLA-binding motifs were further visualized with iceLogo [52,53], and representative 9-mers from IntroSpect and conventional database search were displayed in Figure 2b as having high similarities. We also obtained peptides of the corresponding HLA allele from IEDB [54] and compared them with those obtained by us, and the results showed that the sequence motifs of our datasets were highly consistent with those from IEDB (Figure S9). To quantify the similarities of the HLA-binding motifs, we used the Pearson Correlation Coefficients between the amino acid frequencies (PCC_aaf_) of peptides averaged over all positions. The average PCC_aaf_ of all positions (All) and each anchor position (P2, P3, P9) [55,56,57,58] are all greater than 0.95 (Figure 2c and Figure S7).

Finally, to validate the peptides identified by IntroSpect, we randomly selected a list of peptides for experimental verification from the K562 dataset. A total of 118 peptides (91 peptides identified by both methods and 27 peptides newly identified by IntroSpect proportionally) were synthesized and analyzed under the same MS acquisition conditions as that of K562 cell line. The spectra of synthetic peptides with the highest PSM scores were then compared to the spectra of eluted peptides from K562 cell line in the experiment to confirm or reject the peptide identity. We found that 97.80% of the peptides (89 out of 91) identified by both methods and 96.30% of those (26 out of 27) detected by IntroSpect can only be confirmed by spectral validation (Table 2). Tests on Comet and MaxQuant yielded similar results (Table S1). Collectively, these results demonstrate that IntroSpect can not only identify many more peptides, but also achieve an accuracy that is on par with the conventional search method.

### 3.4. IntroSpect Inherits the Results of Conventional Database Search

**Figure 3:**
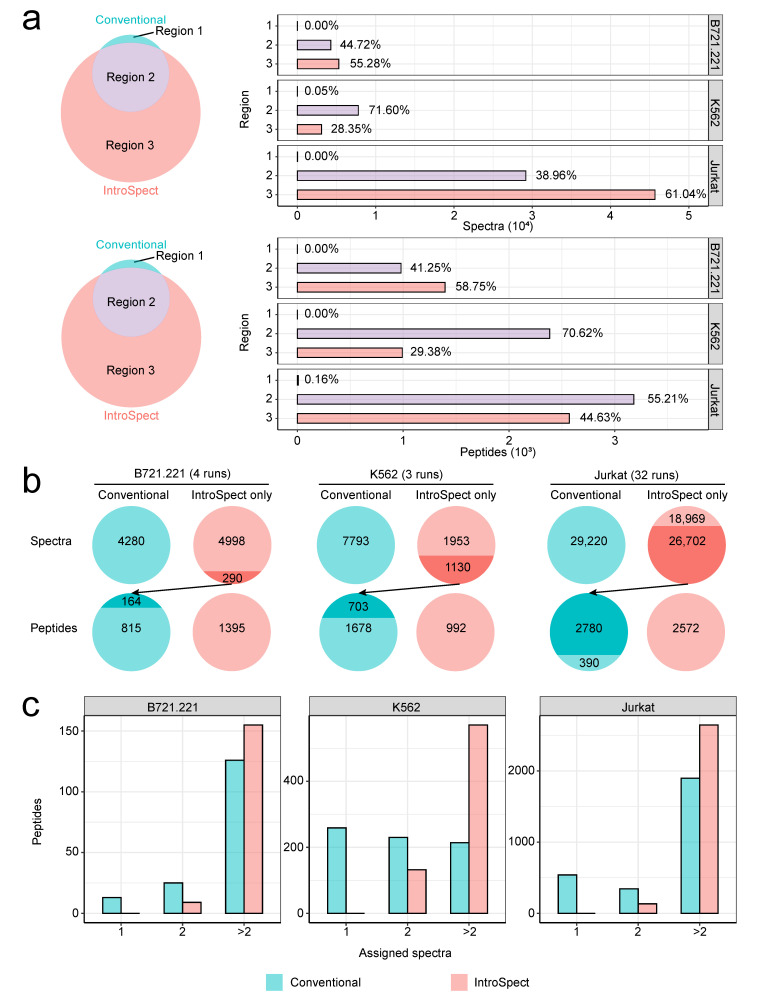
The high consistency of identified spectra and peptides between conventional and IntroSpect search. (**a**) Most of the spectra (top panel) or immunopeptides (bottom panel) detected by conventional method can be identified through IntroSpect. Regions 1, 2 and 3 denote spectra (top panel) or immunopeptides (bottom panel) detected by conventional only, both, or IntroSpect only. The percentages are calculated based on the total number of peptides or spectra identified by both methods. The gray boxes on the right panel denote cell lines. (**b**) A fraction of spectra newly identified by IntroSpect were matched to peptides previously identified by conventional search (refined peptides). These refined peptides were indicated in dark shades. (**c**) The number of assigned spectra for refined peptides increased substantially.

In MS data analysis, spectra provide the raw evidence for identified peptides. Therefore, the essence of newly identified peptides by IntroSpect is a reassignment of the spectra not recognized in conventional search. Based on IntroSpect’s methodology, we hypothesized that the identified spectra and peptides from IntroSpect would cover the vast majority of those from conventional search. Indeed, when we calculated the overlap of both identified spectra and peptides from the two methods, the overlapped spectra or peptides accounted for more than 99% of those identified by the conventional method in three datasets (Figure 3a). Moreover, there were on average 48% of spectra and 44% of peptides identified by IntroSpect alone. We further observed that part of the unique spectra (6 to 58%) identified by IntroSpect matched to peptides (17 to 88%) already identified by conventional search, boosting the evidence of these previously identified peptides, Figure 3b). We call them refined peptides, which are those that can be identified in the conventional search but are assigned extra spectra by IntroSpect. The proportions of refined peptide matched to 1 spectrum, 2 spectra and >2 spectra are 21%, 21% and 58% respectively in conventional search, while the corresponding proportions are 0%, 10% and 90% respectively in IntroSpect search (Figure 3c). Both lines of evidence, i.e., the overlap between IntroSpect and conventional search and the added support of IntroSpect identified spectra for refined peptides, showed the high consistency between these two search strategies and validated our design choice of not simply aggregating different rounds of iterative search, which rendered the extra benefit of a unified global FDR.

### 3.5. The Database Generated by IntroSpect Is Smaller and More Targeted Than SpectMHC

**Figure 4:**
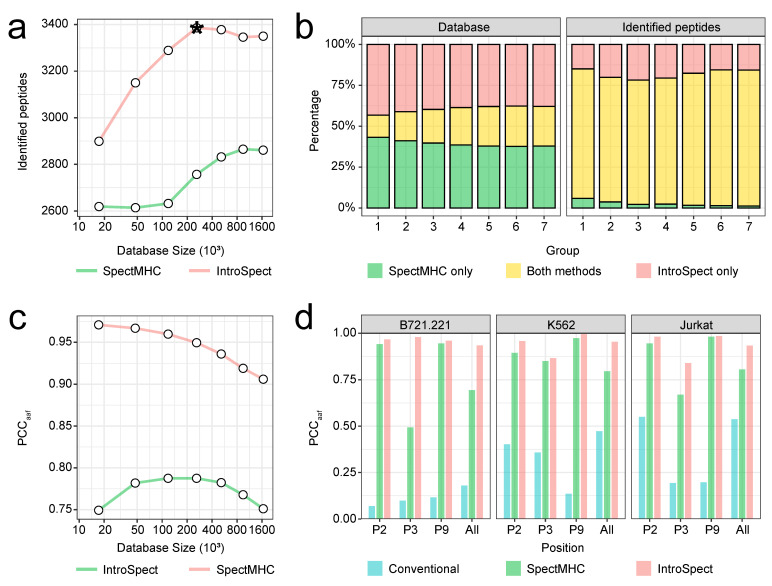
IntroSpect generates a smaller and more targeted database than that of SpectMHC. (**a**) The line plot compares the numbers of identified peptides by SpectMHC and IntroSpect on databases with various matching sizes. The data point with an asterisk corresponds to the motif score of 0.3, the empirically chosen optimal threshold for IntroSpect. (**b**) The bar plot shows the relationship between the databases and identified peptides of IntroSpect and SpectMHC on databases with various sizes. (**c**) The line plot comparing the PCC_aaf_ by SpectMHC and IntroSpect search on databases with various sizes. (**d**) PCC_aaf_ at P2, P3, P9 and all positions between the databases and identified peptides by SpectMHC, IntroSpect and conventional search on the three datasets (B721.221, K562 and Jurkat).

Previous studies have suggested that small, targeted databases are beneficial for MS database search ^15^. Here we have shown that IntroSpect does have a smaller database, and is more sensitive than SpectMHC (Figure 1b,c). However, since IntroSpect learns motifs from the initial search results while SpectMHC learns motifs from external data, we suspect that their targeted databases differ by more than just size. To investigate this, we adjusted the thresholds of IntroSpect and SpectMHC to obtain pairs of target databases with the same size for the K562 cell line, which has been engineered to express a single HLA-A*11:01 allele. All the generated databases of different sizes were used to identify peptides for the K562 dataset, and IntroSpect still had apparent advantages over SpectMHC in terms of the numbers of identified peptides (Figure 4a). Furthermore, although the overlap between the databases by the two methods was small (~20%), the overlap between the identified peptides was large (~80%), and the number of peptides solely identified by IntroSpect was about 10 times more than that by SpectMHC across different database sizes (Figure 4b). Clearly, these results indicate that the database generated by IntroSpect is more targeted, or of higher quality, when used in MS database search, compared with that of by SpectMHC. This is likely because motifs learned from the same MS data (as in IntroSpect) are a better match than those learned from external data (as in SpectMHC). To quantify, we calculated the average PCC_aaf_ at all positions between the peptides in the databases and those identified by SpectMHC or IntroSpect, and IntroSpect has higher PCC_aaf_’s across different database sizes (Figure 4c and Figure S10). We also computed the same quantity across all three datasets with the default thresholds (PSSM score > 0.3 for IntroSpect and NetMHCpan rank < 2% for SpectMHC) of SpectMHC and IntroSpect and observed the same trend (Figure 4d).

### 3.6. IntroSpect Identified More Neoepitopes Than Conventional Method

Having established the superior performance of IntroSpect, we next applied it to a key application in immunology, which is to directly identify neoepitopes from MS profiling of the immunopeptidome. This is a very challenging problem, since neoepitopes are typically of low abundance. However, due to the practical importance of neoepitopes in cancer immunotherapies, great efforts have been made to identify them in the past, going beyond the standard MS techniques, such as manual inspections of MS spectra without stringent FDR filtering [59], or experimentally altering the antigen processing machinery (APM) components to increase the abundance of neoepitopes [60,61,62,63].

**Figure 5:**
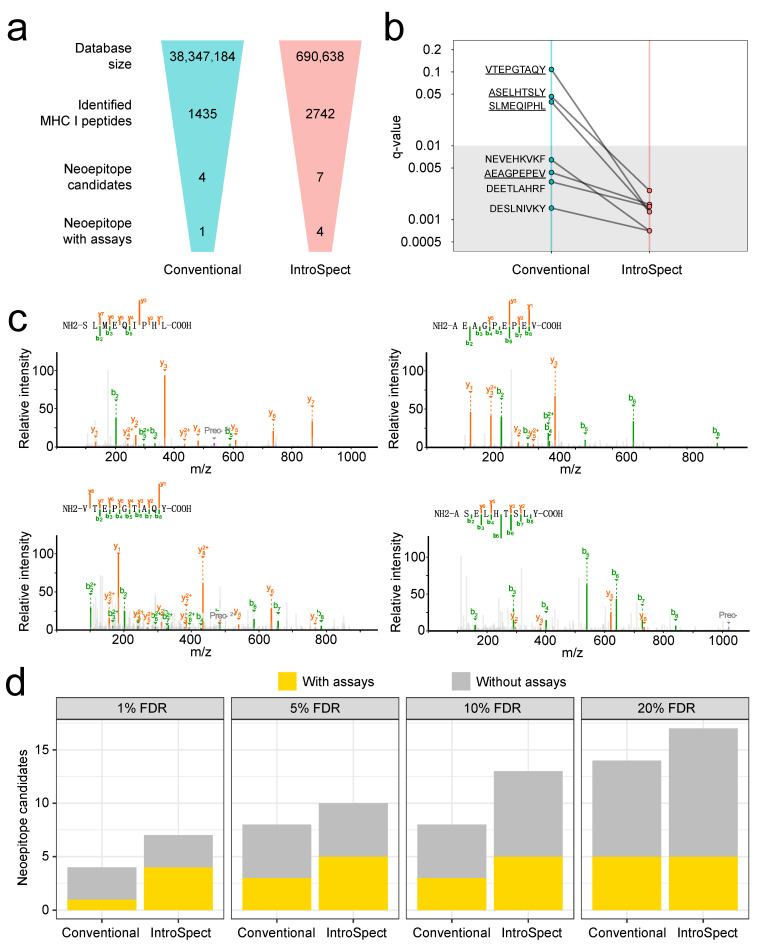
IntroSpect identified more neoepitopes than conventional search. (**a**) Flowcharts indicating key steps involved in neoepitope discovery. (**b**) Percolator q-values of neoepitopes identified by both methods are plotted. Underlined peptides have support in other studies. (**c**) Spectra of neoepitope candidates assigned by IntroSpect with assay support. Peaks represent b ions in green, y ions in orange and precursor ions in dark grey. (**d**) The numbers of neoepitopes identified by the two methods under different FDRs.

Here we generated immunopeptidome, as well as sequencing data for the HCT116 cell line, by standard experimental techniques, and focused on comparing the abilities of conventional search and IntroSpect in identifying neoepitopes. Based on the sequencing data of HCT116, we first generated all 9–11 mer potential neoepitopes and added them to the Uniprot database, and performed a conventional and IntroSpect search, as described previously. As before, IntroSpect was able to identify substantially more peptides than conventional search (2742 versus 1435), but more importantly, 7 neoepitopes were identified by IntroSpect versus 4 by conventional search, about a two-fold increase (Figure 5a, Table S2). As expected, the q-values of these 7 neoepitopes were significantly reduced in IntroSpect, compared with a conventional search (Figure 5b). We also manually inspected the supported spectra of these 7 neoepitopes, and they are all of high quality (Figure 5c and Figure S11). To further examine the quality of these identified neoepitopes, we exhaustedly searched for established experimental evidence of them, including ligand presentation, qualitative binding and IFNg release assay [54]. We were able to find previous evidence for 1 of the 4 neoepitopes identified by conventional search, but all 3 additional neoepitopes discovered by InstroSpect (4 of 7 in total). In addition, we also performed the test by SpectMHC. Its identified peptides were 1/4 less than IntroSpect (2024 versus 2742), and it identified one less neoepitope than IntroSpect (6 versus 7, Figure S13).

Becker et al. recently proposed to use 5AZA to treat the HCT116 cell line to enhance its antigen presentation ability, and identified a number of extra neoepitopes based on this technique [63]. Interestingly, while conventional search with our data was not able to identify any of the neoepitopes discovered by Becker et al., IntroSpect was able to identify two of them (SLMEQIPHL under 1% FDR and QTDQMVFNTY under 5% FDR). When studying neoepitopes, researchers routinely use more relaxed FDRs to obtain more sensitive results. Therefore, we also tested the same strategy in our comparison (Figure 5d). As expected, both methods can discover more neoepitopes with more relaxed FDRs (with the potential cost of higher false positive rates), but more importantly, IntroSpect can discover the same number of neoeptitopes with previous assay support, which indicates more reliable results, with a much lower FDR. For example, all 5 neoepitopes with previous assay support can be discovered by IntroSpect with a 5% FDR, but conventional search needs a 20% FDR to uncover them all. This shows that IntroSpect is valuable in reducing the time and labor cost of experimental validation in neoepitope screening studies.

## 4. Discussion

Currently, high-throughput immunopeptidome profiling is usually based on an MS database search, but the lack of specific digestion leads to low sensitivity. Here, we developed IntroSpect, a motif-guided immunopeptidome database building tool, to overcome this challenge. By testing on diverse immunopeptidome datasets, we showed that IntroSpect could significantly increase the sensitivity of identification compared with not only conventional searches but also a previously developed database building tool, SpectMHC, while maintaining a high accuracy. It is also worth mentioning that it can be easily combined with existing post-processing tools, as well to potentially achieve further performance improvement.

However, IntroSpect is not without limitations. Currently, IntroSpect may only be suitable for improving traditional search engines. For example, we also tested IntroSpect with the popular de novo-assisted database search tool PEAKS [64,65,66], and the improvement is quite limited, with an average of less than 10% (Figure S12). In addition, the current PSSM model is peptide length and HLA allele-specific, which means that the high-confidence peptides identified in the initial search must be further subdivided for model training. When the peptides identified from a conventional search are relatively few, say <500, the training set of a certain length and HLA allele might be too small to effectively train the corresponding PSSM model, and in such cases, SpectMHC could perform better. One way to address this limitation is to adopt deep learning techniques to leverage existing, large scale MS data to pre-train length independent sequence models, and then adapt the pre-trained models to specific experiments by transfer learning, which remains to be our future work. The motif scores, which only serve as an empirically chosen threshold to filter out highly unlikely peptides, could also be better utilized. One way to do so is to assign weighted prior probabilities for different peptides based on their motif scores when doing database search, similar to what has been developed in the constrained de novo sequencing approach by Li et al. [67].

Nonetheless, we believe the simple and effective strategy implemented in IntroSpect has significantly moved the quality of MS profiled immunopeptidome analysis forward, and opened the door to apply this exciting MS technique in broader scenarios, such as in understanding non-canonical or post-translationally modified immunopeptides [68,69].

## Figures and Tables

**Table 1:** Summary of immunopeptidome data sets.

Dataset	Source	Spectra	HLA Alleles
K562	Inhouse	64,572	transduced to express only A*11:01
B721.221	Public ^39^	111,662	transduced to express only A*02:07
Jurkat	Public ^21^	670,119	A*03:01, B*07:02, C*04:01, A*03:01, B*35:03, C*07:02
Train1	Public ^40^	84,453	A*11:01, B*27:02, C*03:03, A*11:01, B*55:01, C*05:01
Train9	Public ^40^	88,437	A*11:01, B*51:01, C*01:02, A*68:01, B*56:01, C*07:02
Train10	Public ^40^	170,101	A*29:02, B*44:03, C*04:01, A*29:02, B*35:01, C*16:01
Train13	Public ^40^	128,712	A*01:01, B*08:01, C*07:01, A*03:01, B*35:01, C*04:01
Train22	Public ^40^	273,039	A*31:01, B*08:01, C*12:03, A*03:01, B*38:01, C*07:01
Train28	Public ^40^	192,712	A*03:01, B*35:03, C*03:03, A*03:01, B*51:01, C*04:01
Train29	Public ^40^	175,619	A*03:02, B*44:03, C*03:03, A*26:01, B*35:02, C*16:01
Train32	Public ^40^	123,863	A*29:02, B*44:03, C*07:02, A*03:01, B*07:02, C*16:01
Train33	Public ^40^	463,383	A*02:03, B*15:02, C*08:01, A*68:01, B*15:13, C*08:01
Train45	Public ^40^	178,449	A*31:01, B*44:02, C*05:01, A*01:01, B*67:01, C*12:03
Train48	Public ^40^	468,069	A*24:02, B*18:01, C*07:02, A*25:01, B*07:02, C*12:03
Train50	Public ^40^	142,681	A*33:03, B*44:03, C*07:06, A*68:01, B*35:01, C*04:01
Train55	Public ^40^	281,891	A*01:01, B*08:01, C*07:01, A*24:02, B*08:01, C*07:01
Train62	Public ^40^	168,243	A*02:01, B*44:02, C*05:01, A*68:01, B*44:02, C*07:04
Train63	Public ^68^	329,221	A*31:01, B*44:02, C*05:01, A*02:01, B*27:05, C*02:02

**Table 2:** Randomly selected peptides identified by IntroSpect and conventional database search were confirmed by spectral validation.

Software	Source	Identified	Selected for Synthesis	Confirmed Positive	Precision (%)
MS-GF+	Both conventional and IntroSpect	2385	91	89	97.80
	IntroSpect only	993	27	26	96.30

## Data Availability

The MS data of K562 and HCT116 datasets have been deposited in the public proteomics repository MassIVE (https://massive.ucsd.edu) accessed on 27 July 2021 with accession number MSV000086567 and MSV000087927. The sequencing data, as well as the above MS data, have also been deposited into the CNGB Sequence Archive (CNSA) [70] of China National GeneBank DataBase (CNGBdb) [71] with the accession number CNP0001446.

## Supplementary Materials

The following supporting information can be downloaded at: https://www.mdpi.com/article/10.3390/biom12040579/s1, Figure S1. IntroSpect decreases the database size and increases the proportion of identified MS/MS spectra with Comet; Figure S2. Peptides identified by IntroSpect, SpectMHC and the conventional search with MaxQuant; Figure S3. The q-value distribution of conventional search and IntroSpect search; Figure S4. The score distribution of conventional search and IntroSpect search; Figure S5. The histogram of predicted BA rank values of peptides identified by the conventional and IntroSpect search with MS-GF+ on more datasets; Figure S6. The histogram of predicted BA rank values of peptides identified by the conventional and IntroSpect search with Comet; Figure S7. Amino acid frequencies at each position of the peptides identified by the conventional and IntroSpect search with MS-GF+; Figure S8. Amino acid frequencies at each position of the peptides identified by the conventional and IntroSpect search with Comet; Figure S9. The sequence logo comparison of immunopeptides in various datasets by conventional search, IntroSpect search and from IEDB; Figure S10. The comparison of PCC_aaf_ at each position between IntroSpect and SpectMHC; Figure S11. Spectra of neoepitope candidates; Figure S12. Peptides identified by IntroSpect, SpectMHC and the conventional search with PEAKS; Figure S13. Neoepitopes identified by IntroSpect, SpectMHC and the conventional search in the HCT116 dataset; Table S1. Randomly selected peptides identified by IntroSpect and conventional database search with Comet and MaxQuant were confirmed by spectral validation; Table S2. The neoepitope candidates identified from HCT116 cell line; Table S3. The effect of clustering number on the performance of IntroSpect.
