# Supplementary material for: IntroSpect: Motif-Guided Immunopeptidome Database Building Tool to Improve the Sensitivity of HLA I Binding Peptide Identification by Mass Spectrometry
Source: Biomolecules. 2022 Apr 14;12(4):579. doi: 10.3390/biom12040579 (PMC9025654; doi:10.3390/biom12040579)
Supplement: Supplementary file 1 [file biomolecules-12-00579-s001.zip › biomolecules-1648366-supplementary.pdf]

IntroSpect: motif-guided immunopeptidome database building tool to improve the sensitivity of HLA I binding peptide identification by mass spectrometry

Supplementary material lists

Supplementary Figure S1. IntroSpect decreases the database size and increases the proportion of identified MS/MS spectra with Comet.

Supplementary Figure S2. Peptides identified by IntroSpect, SpectMHC and the conventional search with MaxQuant.

Supplementary Figure S3. The q-value distribution of conventional search and IntroSpect search.

Supplementary Figure S4. The score distribution of conventional search and IntroSpect search.

Supplementary Figure S5. The histogram of predicted BA rank values of peptides identified by the conventional and In-troSpect search with MS-GF+ on more datasets.

Supplementary Figure S6. The histogram of predicted BA rank values of peptides identified by the conventional and In-troSpect search with Comet.

Supplementary Figure S7. Amino acid frequencies at each position of the peptides identified by the conventional and IntroSpect search with MS-GF+.

Supplementary Figure S8. Amino acid frequencies at each position of the peptides identified by the conventional and IntroSpect search with Comet.

Supplementary Figure S9. The sequence logo comparison of immunopeptides in various datasets by conventional search, IntroSpect search and from IEDB.

Supplementary Figure S10. The comparison of PCCaaf at each position between IntroSpect and SpectMHC.

Supplementary Figure S11. Spectra of neoepitope candidates.

Supplementary Figure S12. Peptides identified by IntroSpect, SpectMHC and the conventional search with PEAKS.

Supplementary Figure S13. Neoepitopes identified by IntroSpect, SpectMHC and the conventional search in the HCT116 dataset.

Supplementary Table S1. Randomly selected peptides identified by IntroSpect and conventional database search with Comet and MaxQuant were confirmed by spectral validation.

Supplementary Table S2. The neoepitope candidates identified from HCT116 cell line.

Supplementary Table S3. The effect of clustering number on the performance of IntroSpect.

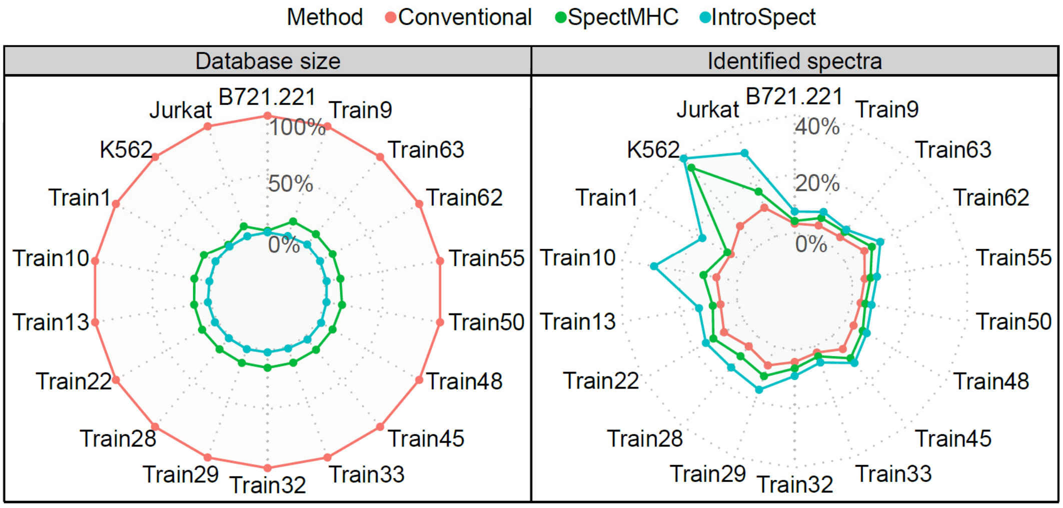

Supplementary Figure S1. IntroSpect decreases the database size and increases the proportion of identified MS/MS spectra with Comet.

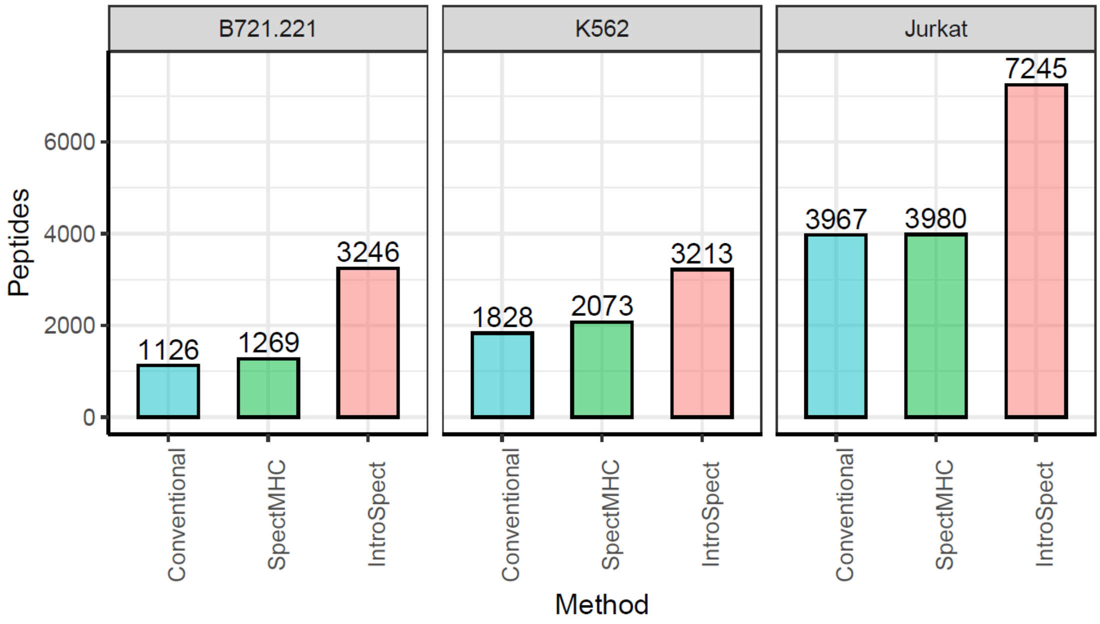

Supplementary Figure S2. Peptides identified by IntroSpect, SpectMHC and the conventional search with MaxQuant.

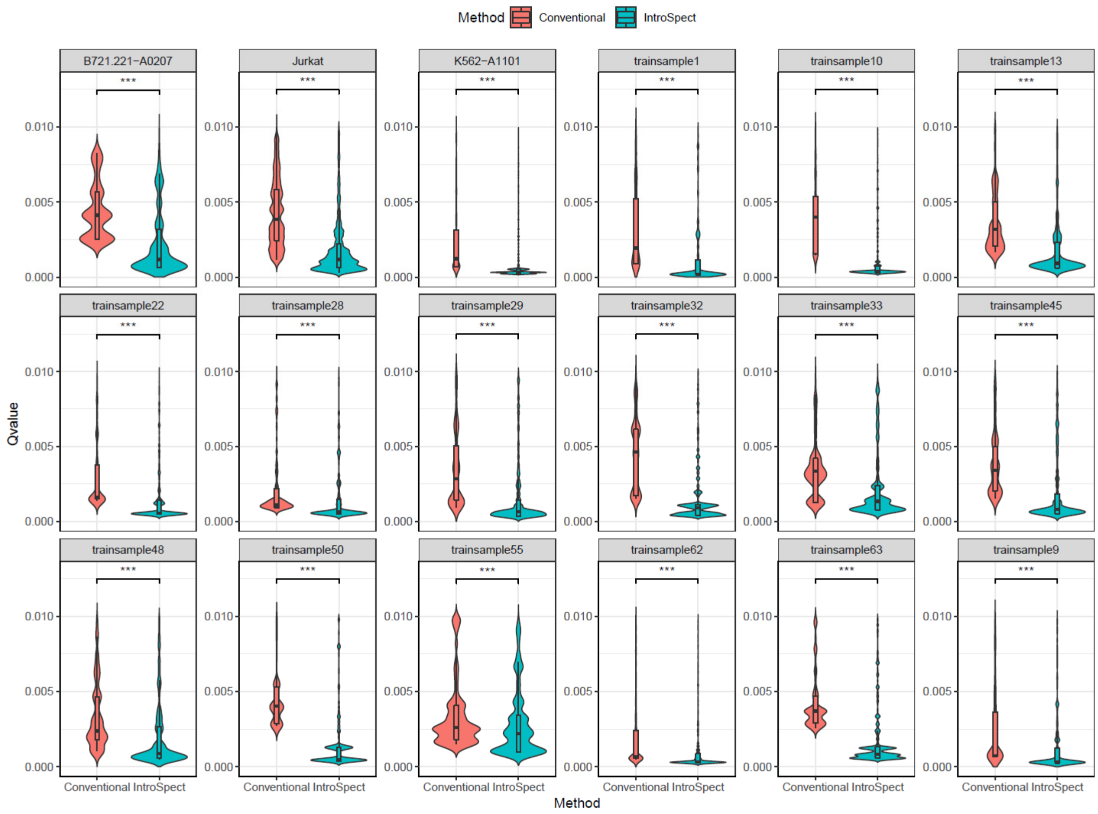

Supplementary Figure S3. The q-value distribution of the conventional search and IntroSpect search with MS-GF+.

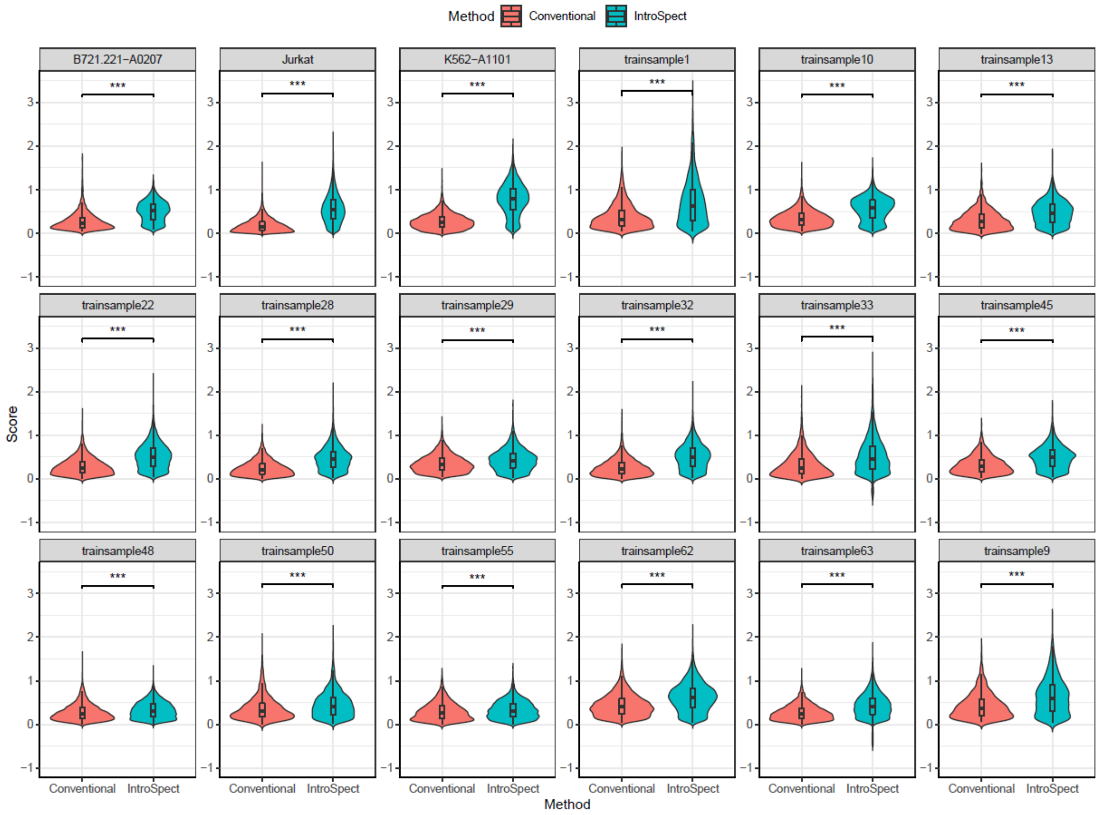

Supplementary Figure S4. The score distribution of the conventional search and IntroSpect search with MS-GF+.

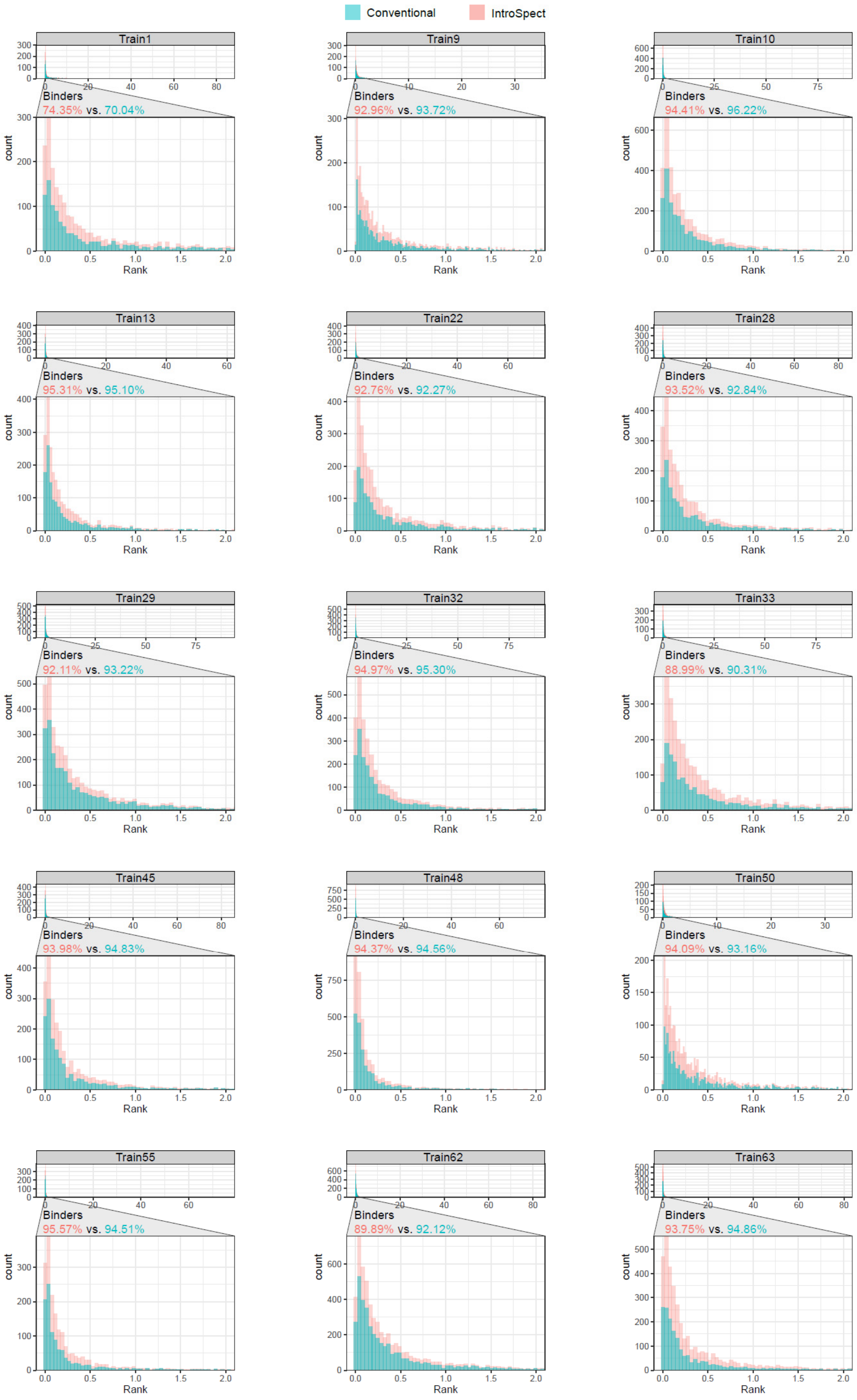

Supplementary Figure S5. The histogram of predicted BA rank values of peptides identified by the conventional and IntroSpect search with MS-GF+.

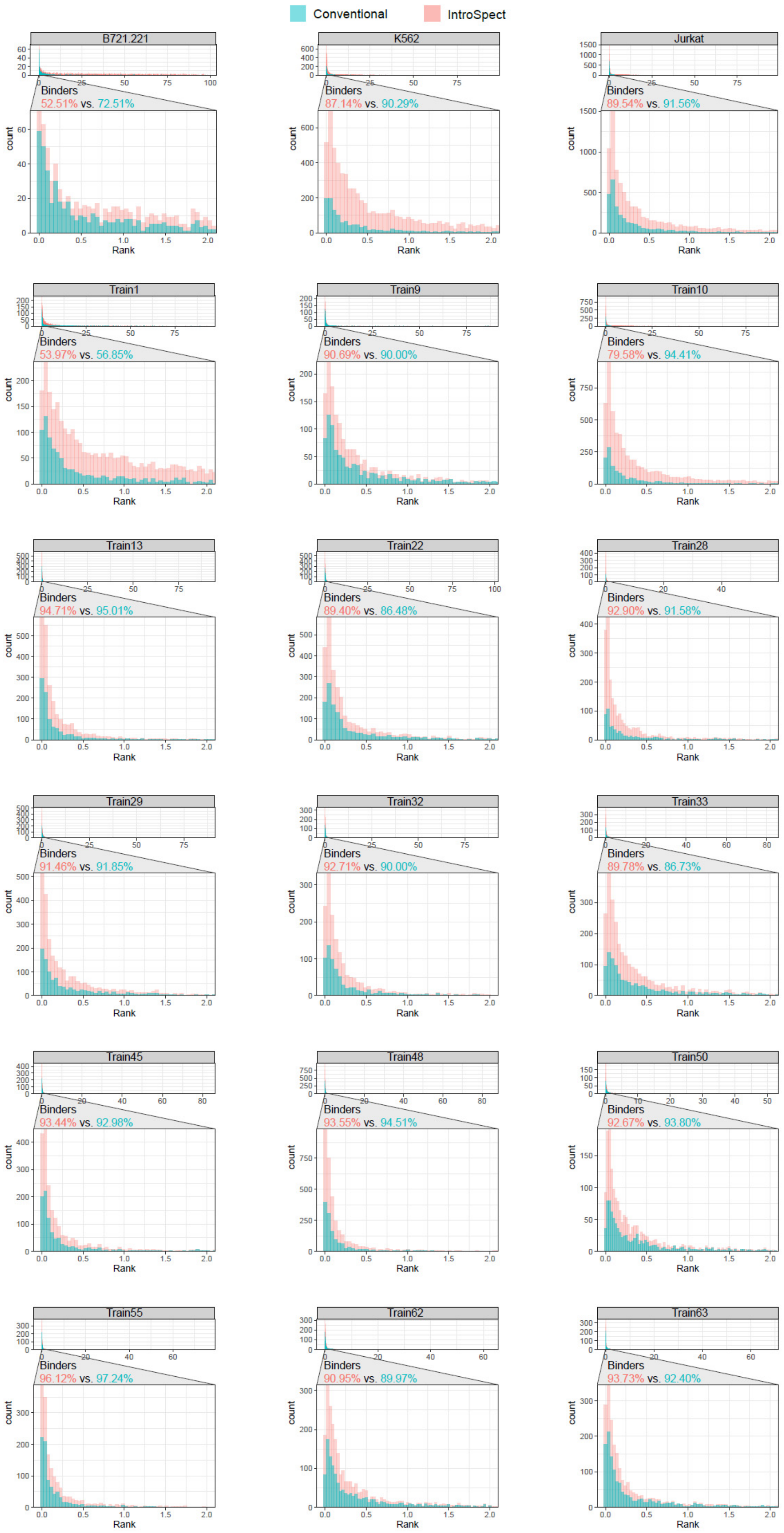

Supplementary Figure S6. The histogram of predicted BA rank values of peptides identified by the conventional and IntroSpect search with Comet.

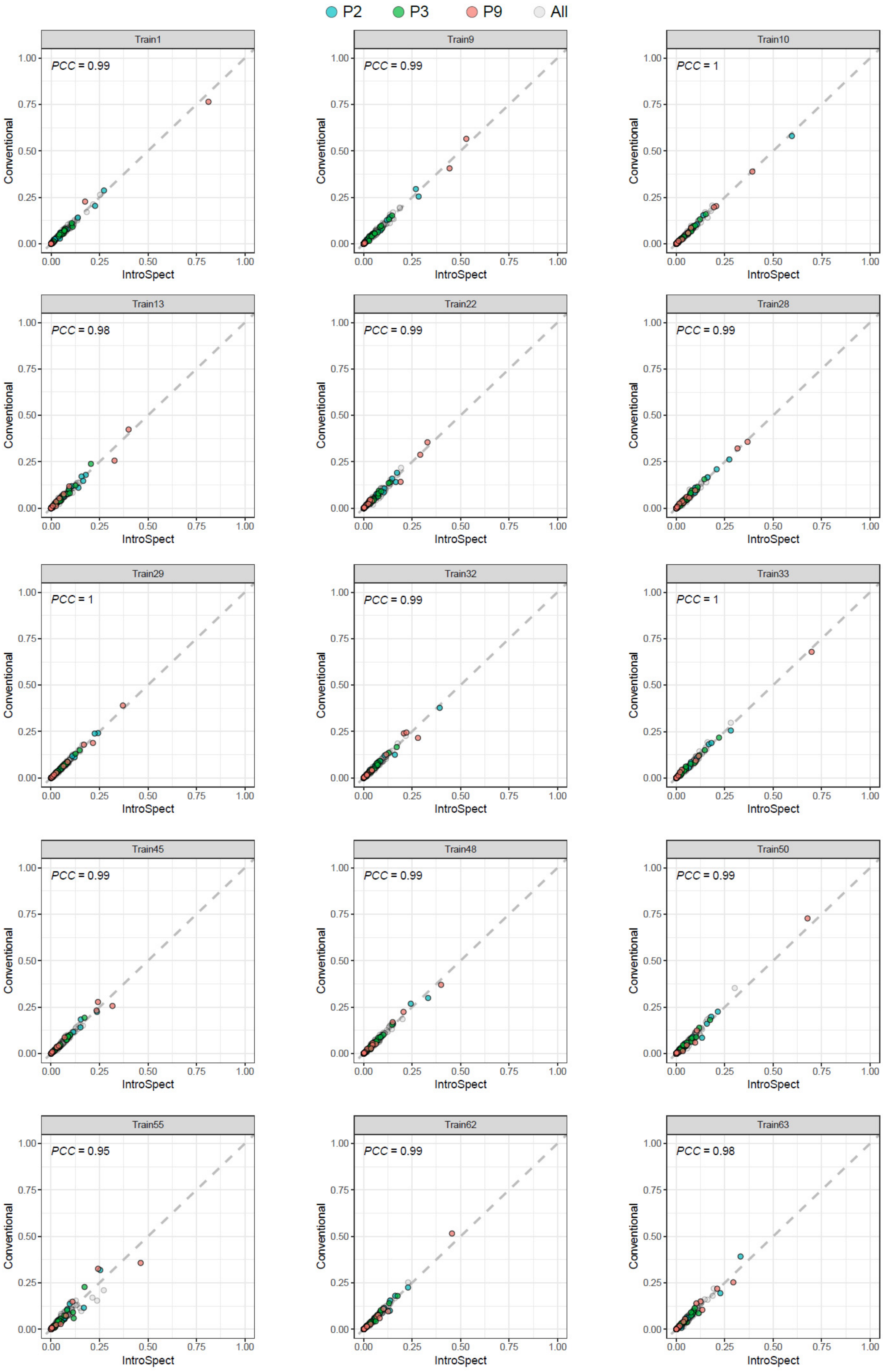

Supplementary Figure S7. Amino acid frequencies of each position within peptides identified by the conventional and IntroSpect search with MS-GF+.

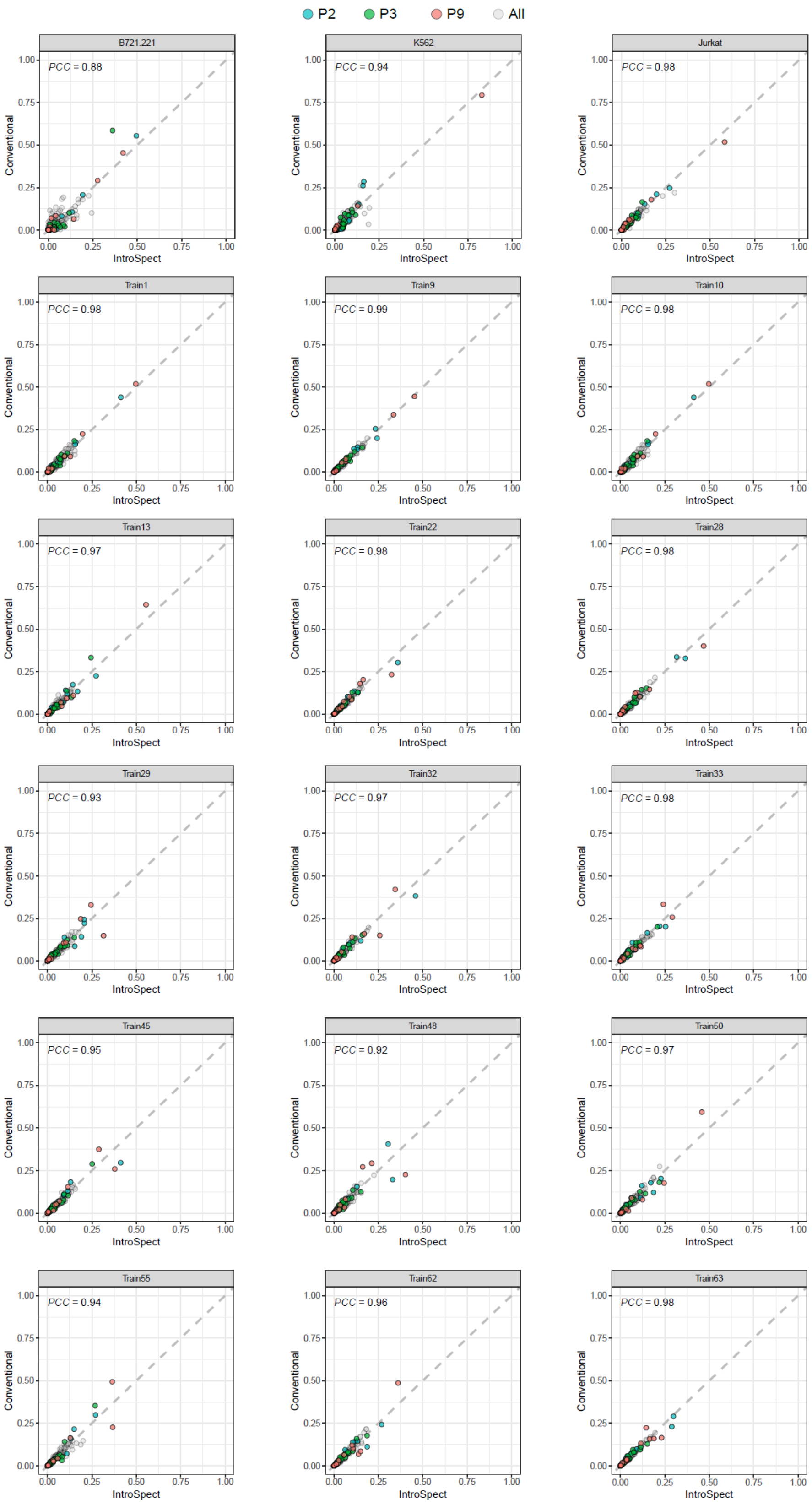

Supplementary Figure S8. Amino acid frequencies of each position within peptides identified by the conventional and IntroSpect search with Comet.

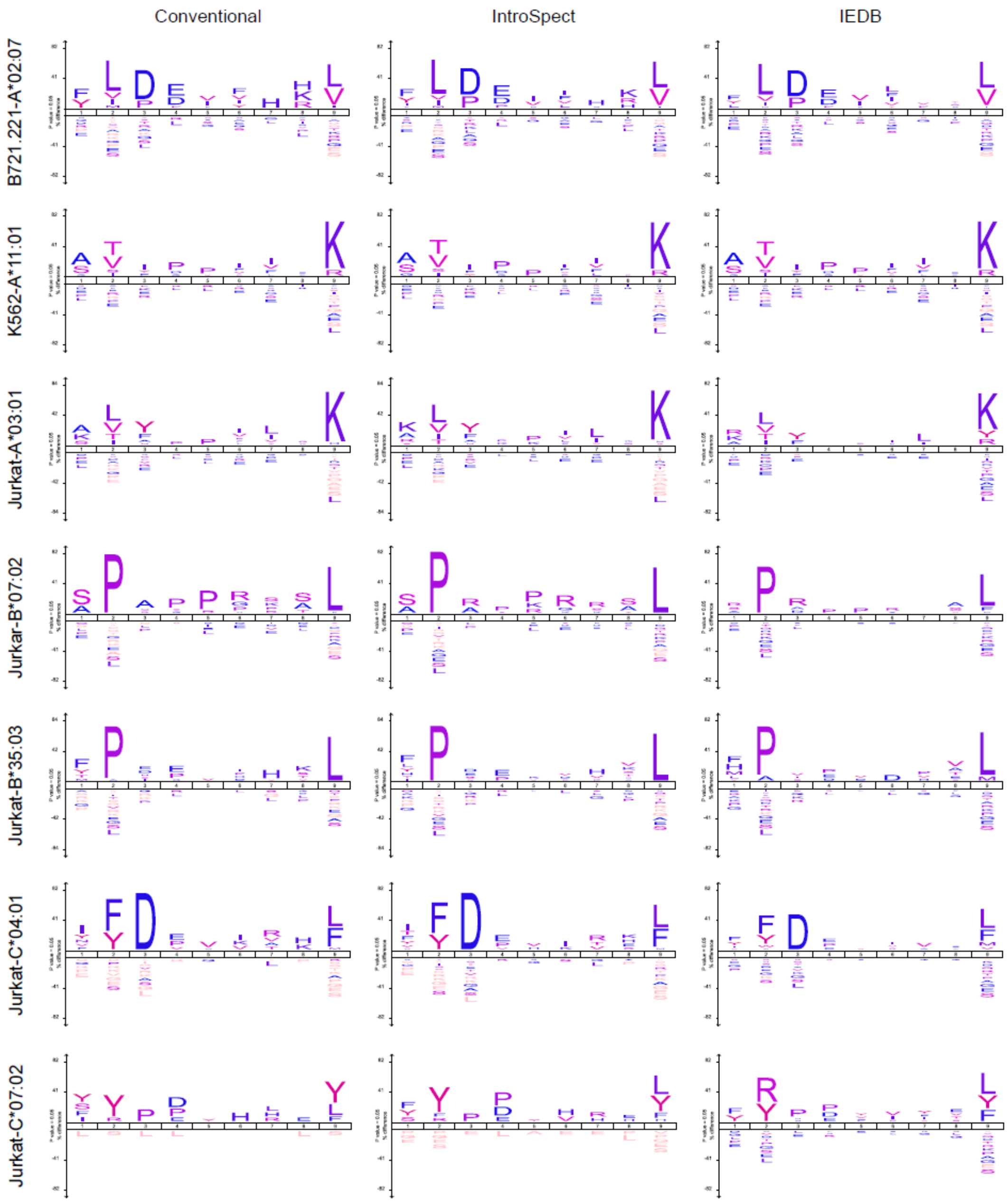

Supplementary Figure S9. The sequence logo comparison of immunopeptides in various datasets by the conventional search, IntroSpect search and IEDB published.

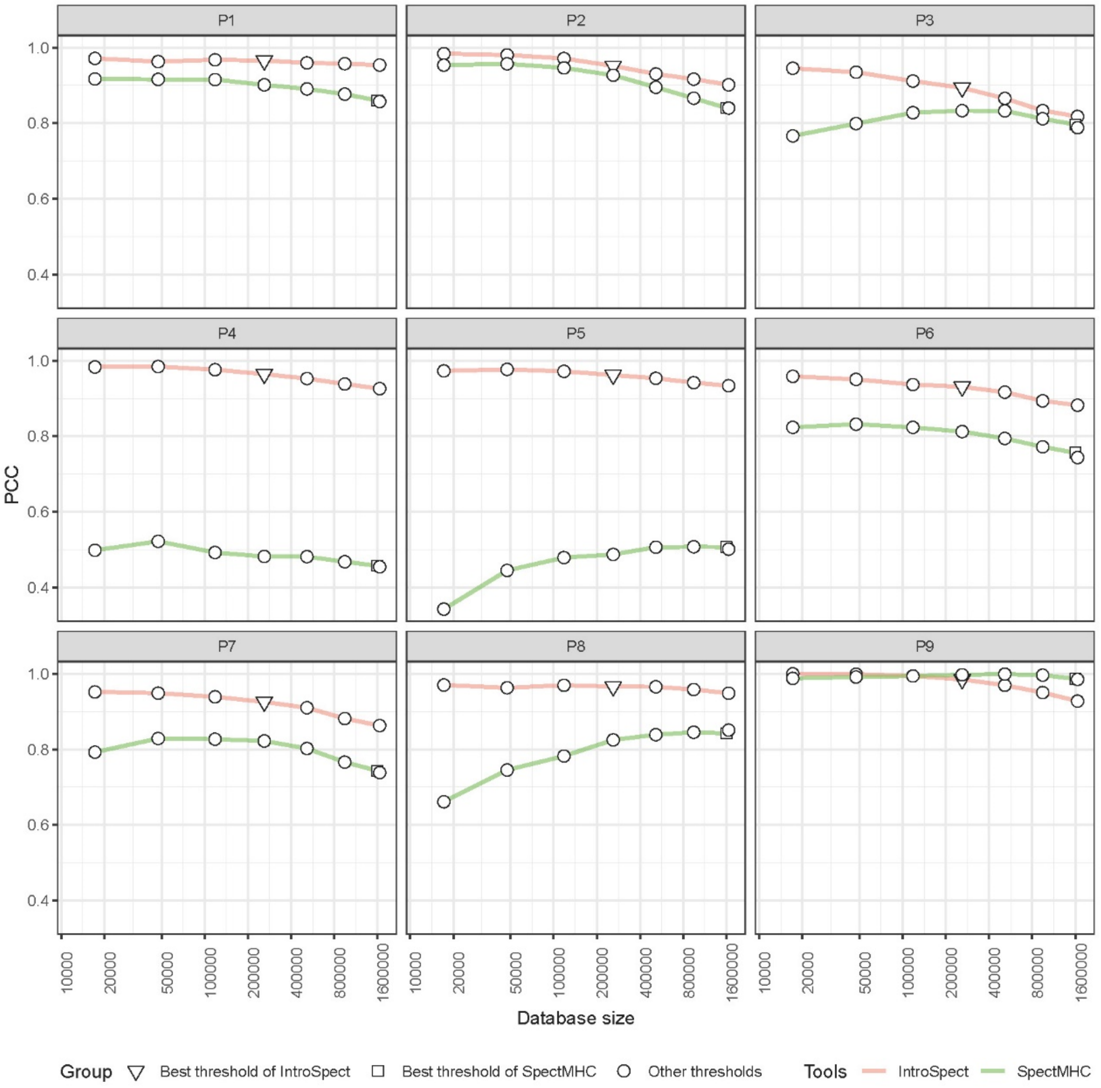

Supplementary Figure S10. The comparison of PCC_{aaf} on each position between the IntroSpect and SpectMHC.

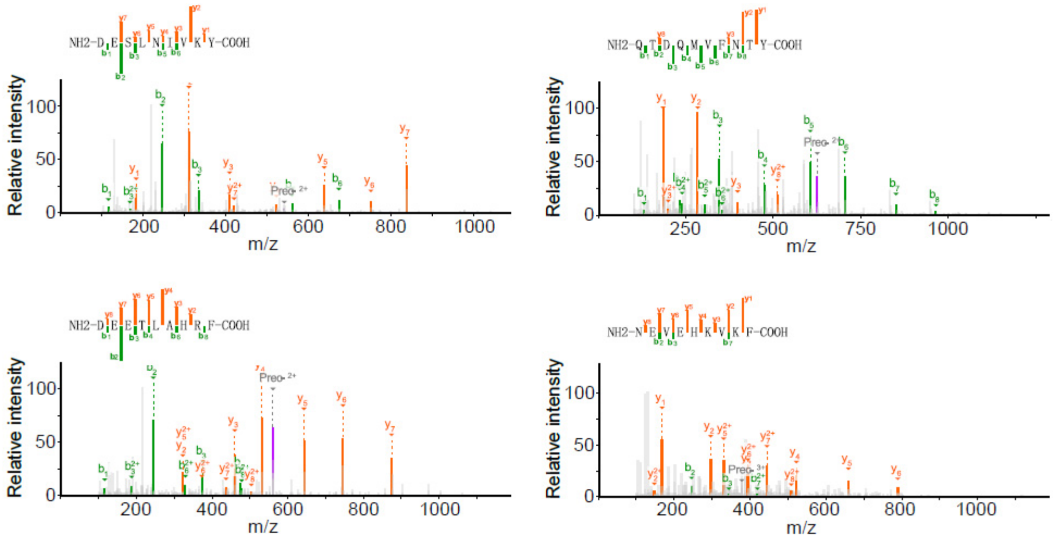

Supplementary Figure S11. Spectra of neoepitope candidates.

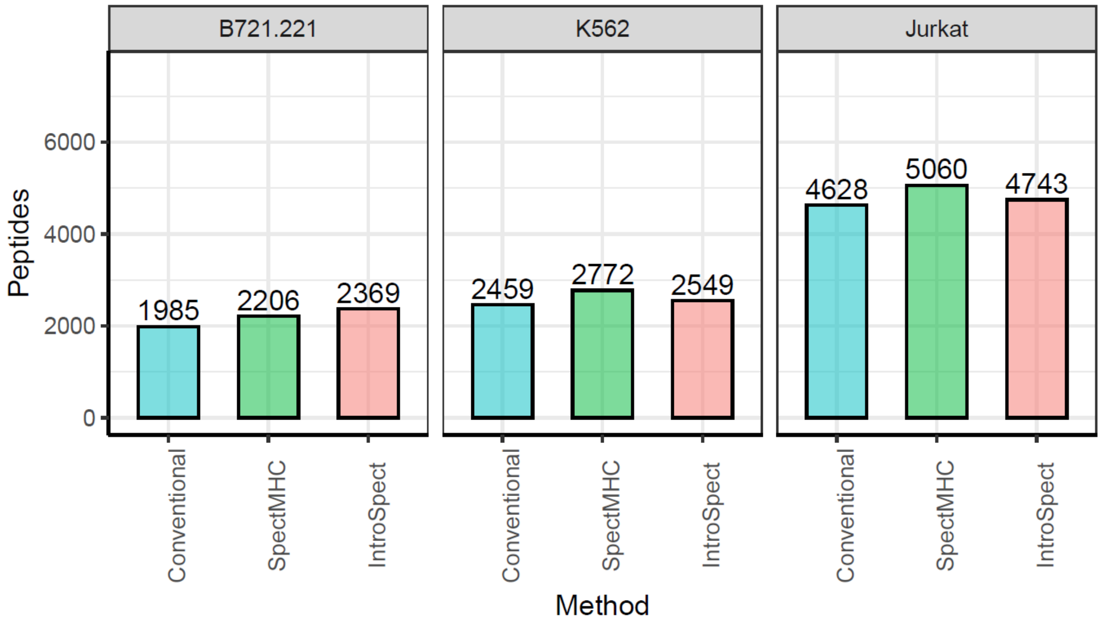

Supplementary Figure S12. Peptides identified by IntroSpect, SpectMHC and the conventional search with PEAKS.

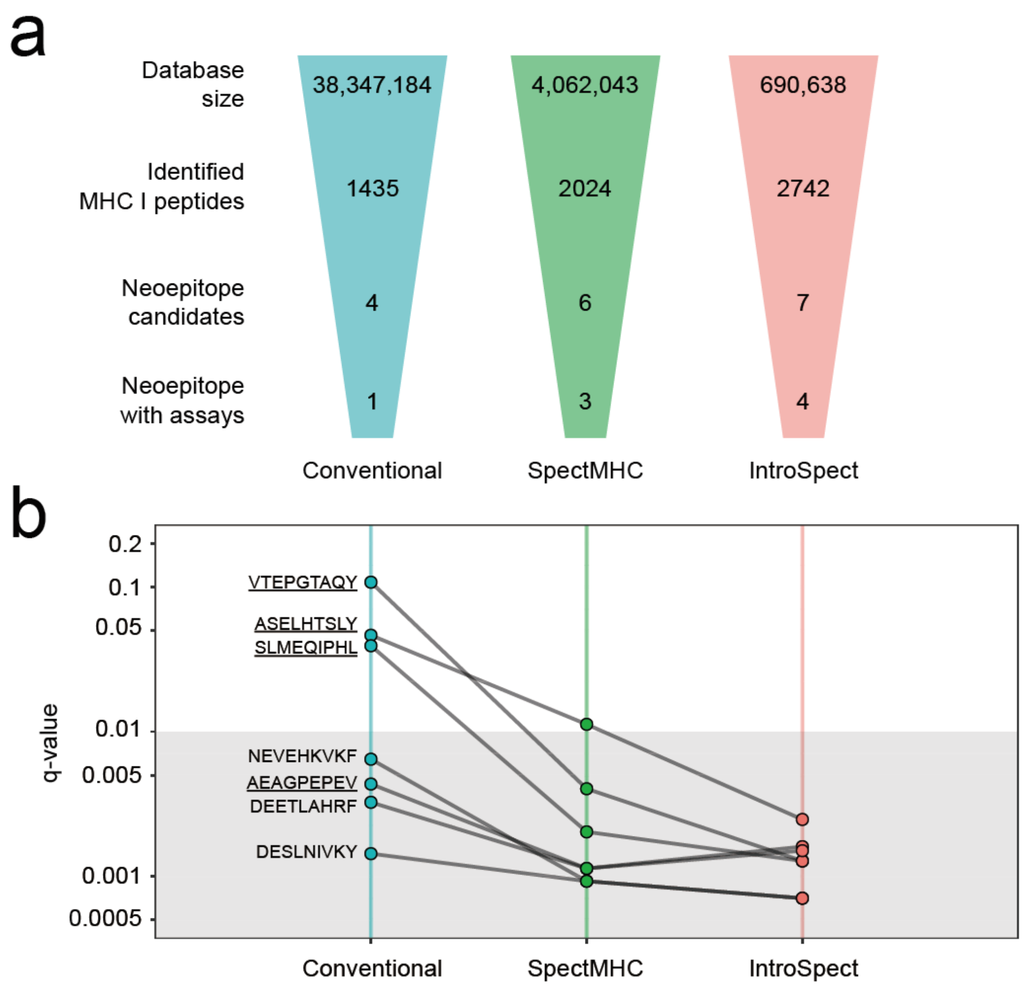

Supplementary Figure S13. Neoepitopes identified by IntroSpect, SpectMHC and the conventional search in the HCT116 dataset. (a) Flowcharts indicating key steps involved in neoepitope discovery. (b) Percolator q-values of neoepitopes identified by all methods are plotted. Underlined peptides have support in other studies.

Supplementary Table S1. Randomly selected peptides identified by IntroSpect and conventional database search with Comet and MaxQuant were confirmed by spectral validation.

Software	Source	Identified	Selected for Synthesis	Confirmed positive	Precision (%)
Comet	Both conventional and IntroSpect	1,360	45	44	97.78
	IntroSpect only	6,548	43	42	97.67
MaxQuant	Both conventional and IntroSpect	1,828	69	69	100
	IntroSpect only	1,389	55	54	98.18

Supplementary Table S2. The neoepitope candidates identified from HCT116 cell line.

ID	Peptide	Gene	Mutation	Site	Expression (TPM)	HLA	Predicted IC50 (nM)	Supporting Evidence
Neo-1	AEAGPEPEV	EIF3B	SNV	p.S64P	58.78	B*45:01	279.57	ligand presentation
Neo-2	VTEPGTAQY	AKAP13	SNV	p.M452T	11.61	A*01:01	24.87	IFNg release
Neo-3	ASELHTSLY	MDN1	SNV	p.H3423Y	14.65	A*01:01	9.43	ligand presentation
Neo-4	SLMEQIPHL	CKAP2	INDEL	p.K603X	18.67	A*02:01	3.59	qualitative binding;<br>ligand presentation; IFNg release
Neo-5	DESLNIVKY	CCZ1B	SNV	p.E71D	15.66	B*18:01	15.83	
Neo-6	DEETLAHRF	CWF19L1	SNV	p.R523H	10.90	B*18:01	39.72	
Neo-7	NEVEHKVKF	SYNE2	SNV	p.I2942V	11.92	B*18:01	25.22	
Neo-8	QTDQMVFNTY	CHMP7	SNV	p.A324T	12.87	A*01:01	15.85	ligand presentation

Supplementary Table S3. The effect of clustering number on the performance of IntroSpect.

Sample	Clustering number	Peptides	Target database size	Predicted binders	Highest KLD in GibbsCluster	Highest sensitivity of identification
trainsample10	1	3,851	223,137	96.22%	yes	yes
	2	3,682	508,793	94.89%		
	3	3,686	749,515	94.57%		
	4	3,631	1,039,755	94.19%		
	5	3,579	1,188,375	94.33%		
	6	3,442	1,463,459	94.36%		
trainsample1	1	2,734	313,314	74.35%	yes	
	2	2,862	600,118	72.42%		
	3	2,897	849,654	72.19%		
	4	2,978	1,105,316	71.67%		yes
	5	2,952	1,439,230	72.89%		
	6	2,933	1,729,313	73.33%		
trainsample13	1	2,252	315,429	95.25%		
	2	2,457	476,518	95.31%	yes	yes
	3	2,449	731,325	95.18%		
	4	2,412	821,779	94.90%		
	5	2,424	919,252	96.74%		
	6	2,403	1,243,004	95.05%		
trainsample22	1	2,892	312,793	92.22%		
	2	3,110	625,447	92.76%	yes	
	3	3,214	874,518	92.59%		
	4	3,258	1,117,899	92.88%		
	5	3,371	1,202,371	92.91%		
	6	3,385	1,301,864	92.82%		yes
trainsample28	1	2,520	370,835	92.98%		
	2	2,856	558,709	93.52%	yes	yes
	3	2,802	783,273	93.79%		

	4	2,772	1,033,559	93.76%		
	5	2,839	1,289,132	93.48%		
	6	2,840	1,363,366	94.19%		
trainsample29	1	3,937	331,612	92.20%	yes	yes
	2	4,054	466,774	91.98%		
	3	4,069	762,425	92.11%		
	4	4,008	937,662	91.99%		
	5	4,007	1,137,703	91.61%		
	6	4,010	1,232,852	91.70%		
trainsample32	1	2,925	351,422	93.91%	yes	yes
	2	3,397	517,652	94.32%		
	3	3,423	936,824	94.97%		
	4	3,393	1,232,594	94.75%		
	5	3,283	1,504,020	94.73%		
	6	3,313	1,593,733	94.63%		
trainsample33	1	3,279	216,554	88.99%	yes	yes
	2	3,234	531,904	89.33%		
	3	3,229	740,573	90.31%		
	4	3,184	922,064	90.33%		
	5	2,909	1,036,538	90.13%		
	6	2,762	1,581,585	89.36%		
trainsample45	1	2,395	389,605	93.86%	yes	yes
	2	2,622	644,495	94.13%		
	3	2,656	884,236	94.62%		
	4	2,677	1,068,221	93.98%		
	5	2,669	1,196,005	94.87%		
	6	2,654	1,166,620	94.05%		
trainsample48	1	3,991	381,131	94.61%		yes
	2	4,050	681,386	94.37%		
	3	3,791	861,730	95.23%		

	4	3,913	1,098,827	94.07%	yes	
	5	3,820	1,485,346	94.19%		
	6	3,763	1,932,503	93.62%		
trainsample50	1	2,757	246,051	91.88%		
	2	2,759	493,886	94.09%	yes	yes
	3	2,627	708,238	93.76%		
	4	2,610	1,088,199	92.45%		
	5	2,553	1,438,081	92.60%		
	6	2,550	1,213,857	92.55%		
trainsample55	1	1,894	258,605	94.61%		
	2	1,912	485,858	95.45%		
	3	1,987	508,586	95.57%	yes	
	4	2,009	506,461	95.57%		yes
	5	1,933	508,238	95.81%		
	6	1,884	577,765	95.38%		
trainsample62	1	6,476	293,167	84.96%		yes
	2	6,368	459,030	88.85%		
	3	6,174	679,822	89.89%	yes	
	4	6,198	883,886	89.72%		
	5	6,217	1,062,362	89.56%		
	6	6,112	1,346,370	89.59%		
trainsample63	1	3,278	322,154	92.98%		
	2	3,742	507,679	93.69%		
	3	3,922	738,594	93.75%	yes	
	4	4,041	910,363	93.07%		
	5	4,042	1,019,680	93.15%		yes
	6	3,805	1,393,341	93.04%		
trainsample9	1	3,170	247,568	92.95%	yes	yes
	2	3,066	428,924	93.44%		
	3	2,851	755,969	92.84%		

4	2,836	1,065,080	93.16%
5	2,667	1,317,339	92.80%
6	2,597	1,458,010	93.11%
